# Global bee bioregions mirror those of other taxa despite a uniquely bimodal latitudinal diversity gradient

**DOI:** 10.1073/pnas.2620558123

**Published:** 2026-10-29

**Authors:** Léo Laborieux, Lena Heinrich, Matheus Januario, Nathan J. Sanders, Thais Vasconcelos, Anshuman Swain

**Affiliations:** ^a^ Department of Ecology and Evolutionary Biology, University of Michigan, Ann Arbor, MI 48109; ^b^ Museum of Paleontology, University of Michigan, Ann Arbor, MI 48108; ^c^ Institute for Global Change Biology, University of Michigan, Ann Arbor, MI 48109; ^d^ Museum of Zoology, University of Michigan, Ann Arbor, MI 48108; ^e^ University of Michigan Herbarium, University of Michigan, Ann Arbor, MI 48108; ^f^ Center for the Study of Complex Systems, University of Michigan, Ann Arbor, MI 48109

**Keywords:** Bees (Anthophila), biogeographic regionalization, phylogenetic beta diversity, arthropod phylogeography, biodiversity gradients

## Abstract

Bioregionalization, the partitioning of the globe into areas of biotic similarity, is one of nature’s most striking patterns, and highly congruent boundaries have been identified across plants, tetrapods, ants, and butterflies. However, these taxa also share a unimodal latitudinal diversity gradient (LDG), raising the question of whether bioregionalization and the LDG are mechanistically coupled or instead arise from largely independent processes acting on different timescales. Bees (Hymenoptera: Anthophila) provide a natural test, their diversity peaking at mid-latitudes rather than in the tropics. Using range estimates for 3,370 species and a phylogenetic beta-diversity framework, we recover 21 bee bioregions nested within at least six major realms (Australasia, Indomalaya-Oceania, Nearctic, Neotropics, Palearctic, and Sub-Sahara) that closely align with those of other taxa despite bees’ atypical diversity gradient. We propose that bioregionalization and species richness are decoupled because they arise from distinct processes: the former reflects historical dispersal and evolutionary history, the latter local diversification, extinction, and environmental filtering. Our analysis provides a global biogeographic framework for future bee research and highlights the processes shaping the world‘s major biogeographic boundaries.

Alfred Russel Wallace published the most influential early map of global vertebrate zoogeographic regions in 1876 (1), building on the six-region scheme Sclater proposed for bird distributions (2). Subsequent work unveiled remarkable biogeographic similarity between tetrapods (3), plants (4), ants (5), and butterflies (6), groups that also share a unimodal LDG peaking in the tropics. This cross-taxon congruence has been read as evidence that bioregionalization and the LDG are coupled, as predicted by the tropical conservatism and “out-of-the-tropics” diversification models (7, 8). Yet the apparent generality of bioregionalization may be more parsimoniously explained by deep-time dispersal and vicariance, which operate similarly across taxa and independently from biodiversity gradients. Bees (Hymenoptera: Anthophila; ~26,000 estimated species) provide an ideal test, their diversity peaking in xeric and seasonally warm temperate regions rather than the wet tropics (9). Here, we generate species range estimates and apply a phylogenetic turnover framework to delineate global bee bioregions using 3,370 species with both spatial and phylogenetic data. We recover bioregions and realms impressively congruent with those of ants, butterflies, tetrapods, and plants (3–6) despite bees’ unusual richness gradient, suggesting biotic turnover shaped by evolutionary history is largely decoupled from local species richness.

Our analysis recovered 21 bee biogeographic regions clustering into at least six major realms (Fig. 1). The northernmost bee assemblages separate into Nearctic (987 species analyzed out of 3,370 in the bioregionalization dataset) and Palearctic (1,180 species) realms, a split shared with tetrapods and plants but unique among insects, contrasting with the Holarctic distributions of ants (5) and butterflies (6). All sub-Saharan bee communities map to a single realm (382 species), whereas Madagascar is traditionally distinct in ant, plant, and tetrapod bioregionalizations; we nonetheless note that our dataset includes only a fraction of the island’s ~240 described bee species, dominated by Halictidae and Apidae rather than Colletidae (10). Bees’ Indomalayan-Oceanian realm (268 species) comprises a single region, contrasting with both the regional subdivision of ant and plant assemblages and the realm-level split at Wallace’s line in tetrapods (3, 6). By contrast, the Australasian and Neotropical realms (299 and 930 species, respectively) have similar boundaries across taxa. Tropical America is the most phylogenetically isolated region (Fig. 1*B*), likely reflecting the deep divergence of Gondwanan-relict lineages from their Old-World relatives rather than the remarkable, but comparatively recent radiations of some neotropical tribes like Meliponini and Euglossini (11). The NMDS and dendrogram (Fig. 1 *B* and *C*) show clear separation among realms relative to within-realm variation, supporting the distinctness of the inferred areas.

**Fig. 1.**
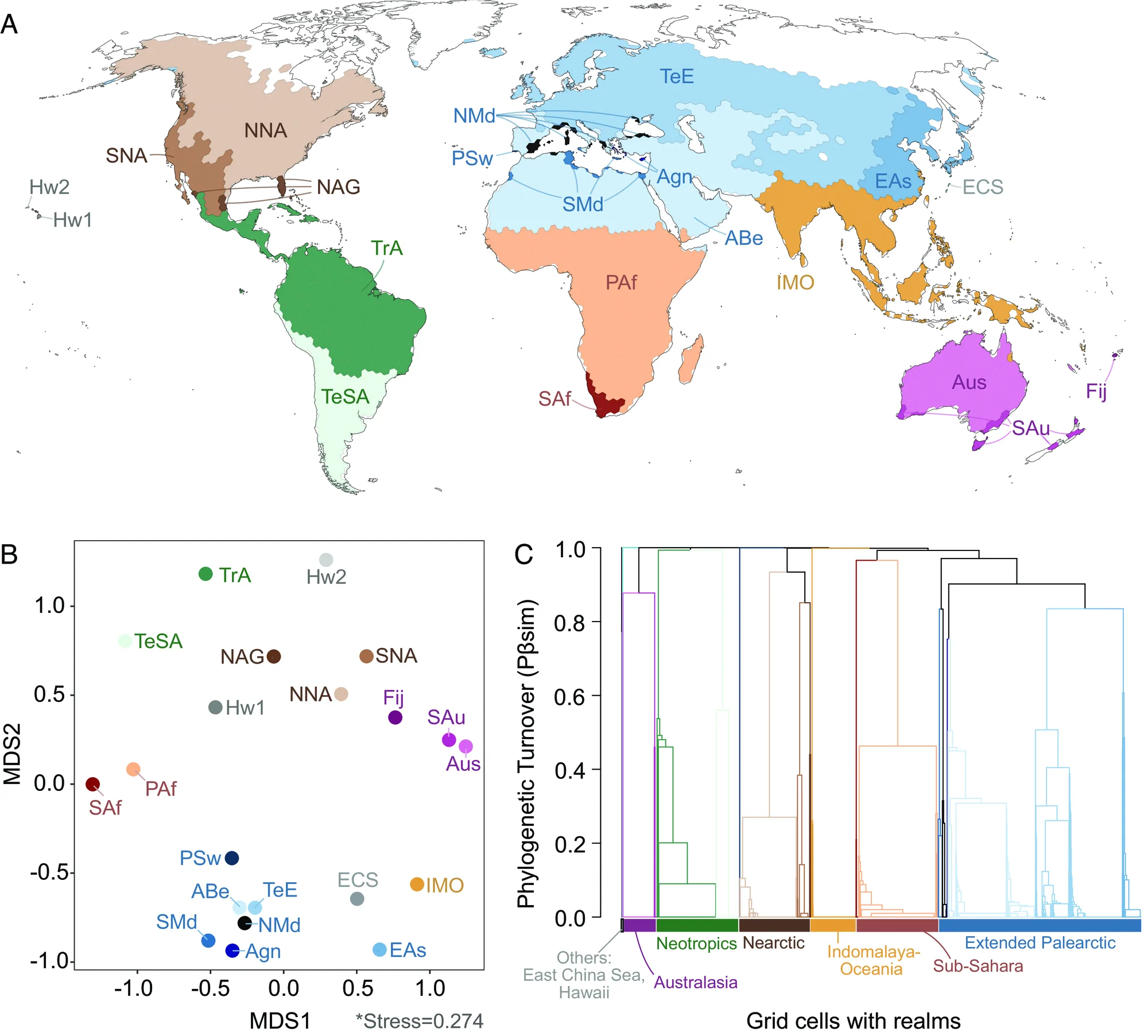
Global bee bioregionalization. (*A*) Bioregions colored by realm (pink: Australasian; yellow: Indomalayan-Oceanian; blue: Extended Palearctic; orange: Sub-Saharan; brown: Nearctic; green: Neotropics). (*B*) NMDS of relative interregion distances. (*C*) Hierarchical clustering dendrogram based on phylogenetic turnover (Pβsim). Abbreviations: Aus: Australia; SAu: South Australasia; Fij: Fiji; TeSA: Temperate South America; TrA: Tropical America; NAG: North America Gulf; SNA: Southern North America; NNA: Northern North America; SAf: Southern Africa; PAf: Pan-African; PSw: Portuguese Southwest; ABe: Arid Belt; TeE: Temperate Eurasia; SMd: South Mediterranean; NMd: North Mediterranean; Agn: Aegean; IMO: Indomalaya-Oceania; EAs: Eastern Asian; Hw1: Hawaii 1; Hw2: Hawaii 2; ECS: East China Sea.

The congruence of bee bioregions with those of other taxa, despite fundamentally different diversity gradients, is suggestive of decoupled drivers. Species richness reflects in situ diversification, local extinction, and ecological filtering; bioregionalization is driven primarily by historical dispersal and evolutionary history, largely independent of contemporary drivers like primary productivity or habitat structure. Bees, which are uniquely diverse in mid-latitude xeric habitats, support this interpretation: the same historical barriers (Atlantic Ocean, Tethys legacy, Saharan arid zone, Central American Seaway) that structure other taxa also delimit bee faunas, producing a remarkably conserved bioregionalization defined by dispersal constraints shared broadly across the tree of life.

Several bee-specific patterns merit further investigation. The apparent distinctiveness of Southern North America may reflect at least two nonexclusive processes, namely i) the exceptional Cenozoic radiation of xeric-adapted lineages such as *Perdita* [~700 species, among the fastest-radiating bee genera; (12)], and ii) this region’s position at a crossroads of Nearctic, Palearctic, and Neotropical faunas under a climate that independently bolsters bee diversity (9). Because Pβsim-based clustering cannot easily differentiate deep, geographically localized radiations from the inflated distinctness generated by faunal admixture at such a convergence zone, disentangling these signals is a priority for future work. The extended Palearctic coherence across North African and Central Asian arid zones (ABe, NMd, SMd, Agn, PSw, TeE) is similarly unrecognized in prior bioregionalizations and may reflect shared evolutionary history of xeric-adapted lineages. Species-poor islands (Fij, ECS, Hw1, Hw2) show low overlap with continental areas, potentially inflating Pβsim and producing artificial clusters; their recognition as region-level entities should therefore be treated as provisional. Many bee lineages are also constrained by the distribution and phenology of their host plants, and future comparison with angiosperm-lineage-level bioregionalizations (4) could clarify how much floral resource availability shapes the boundaries identified here. Closing these data gaps, alongside targeted phylogenetic and biogeographic studies of key radiations, is the most pressing agenda for advancing our understanding of global bee biogeography.

## Materials and Methods

To estimate the biogeographic regionalization of bees, we adapted the framework used by Wang et al. (5) in ants. We compiled bee occurrence data from the BeeBDC global dataset [(13); >18.3 million records spanning all seven bee families]. Species distribution models (SDMs) were fitted for species with sufficient (≥5) occurrence records; minimum convex polygons were used for data-deficient taxa. Predicted distributions were intersected with a global equal-area grid to generate a presence–absence matrix. Phylogenetic turnover (Pβsim, the Simpson component of phylogenetic Sørensen dissimilarity) between grid pairs was quantified across 100 bootstrap trees from a time-calibrated phylogeny (14); cell-wise median dissimilarity was retained to account for phylogenetic uncertainty. Average-linkage hierarchical clustering and a bootstrap-stability procedure identified the optimal number of regions; NMDS ordination visualized interregional relationships (*SI Appendix*).

## Supplementary Material

Appendix 01 (PDF)

## Data Availability

Code and data for bioregionalization analysis, species distribution models data have been deposited in Zenodo (https://doi.org/10.5281/zenodo.20525239) (15).
