# Supplementary material for: Global bee bioregions mirror those of other taxa despite a uniquely bimodal latitudinal diversity gradient
Source: Proc Natl Acad Sci U S A. 2026 Oct 29;123(44):e2620558123. doi: 10.1073/pnas.2620558123 (PMC13732035; doi:10.1073/pnas.2620558123)
Supplement: Supplementary file 1 [file pnas.2620558123.sapp.pdf]

SUPPLEMENTAL INFORMATION (METHODS)

All analyses were performed in R (v4.5.3), and plots were generated using the ggplot2 package (1). The phylogenetic turnover analysis pipeline employed in this study closely follows code previously published by Wang et al. (2).

Species range estimates

We sourced bee occurrences from a dataset published by Dorey et al. (3). This compilation, which combines large, public repositories and more specialized data sources, represents the most complete bee occurrence dataset to date. We filtered the uncleaned dataset to retain only records with species-level identifications and valid coordinates. After filtering, 13,562,193 records spanning 13,230 species were kept for species range estimation.

We estimated individual species ranges using two parallel workflows to accommodate differences in species representation within the dataset. For taxa represented by 5 or more records, we fitted species distribution models (SDMs) using the ENMevaluate function of the ENMeval R package (4). SDM rasters were calculated with 1000 randomly sampled pseudo-absence points based on Worldclim climate data. Output rasters were subsequently converted to binary presence maps by encoding as presence all pixels falling within the top 10% of habitat suitability values. The resulting presence/absence maps were then cropped with a 15-degree buffer around the empirical occurrence bounding box, defined from the minimum and maximum latitude and longitude coordinates of the occurrence records. For all other species, we traced minimum convex hulls encompassing observed occurrences and added a 30 km buffer. The final distribution dataset comprised 6,048 binarized species distribution models and 5,598 distributional polygons, totaling 11,646 bee species. All bee distributional models and range polygons generated for this study are available alongside analysis scripts at https://doi.org/10.5281/zenodo.20525239.

Global bee assemblages and phylogeny

Reconstructing the world's bee assemblages. We projected all binary species ranges on an equal-area hexagon grid ($5 \times 10^4 km^2$, Robinson projection), recording species as present on grid cells that presented any overlap with their distribution raster or polygon. Grid cells comprising fewer than 5 species were excluded to avoid biases potentially introduced by small sample sizes in dissimilarity analyses. The resulting dataset comprised 396,703 bee presence records distributed over 7,035 grid cells.

Bee phylogeny. We sourced a recent molecular phylogeny of Anthophila (5), which represents >80% of currently recognized generic diversity among bees. The taxonomic content of the phylogeny and presence matrix were harmonized, leaving 3,370 bee species for the biogeographic analyses.

Bioregionalization framework

Phylogenetic turnover. Pairwise phylogenetic turnover between all hexagonal cells was quantified as $P\beta_{sim}$, the Simpson component of phylogenetic Sørensen dissimilarity, computed using the phylobeta function of the betapart R package (6). $P\beta_{sim}$ captures species replacement independently of differences in species richness, making it better suited to identifying biogeographic boundaries than total dissimilarity indices. To propagate phylogenetic uncertainty, the analysis was repeated across each of the 100 bootstrap phylogenetic trees from

(5) and the per-cell-pair median Pβsim value across trees was used as the final dissimilarity estimate.

Hierarchical clustering and optimal regionalization. Seven hierarchical clustering algorithms (average, centroid, Ward's D2, single-linkage, complete-linkage, McQuitty, and median) were evaluated by comparing the cophenetic correlation coefficient and Gower distance between the input dissimilarity matrix and the resulting cophenetic distances; average-linkage (UPGMA) consistently produced the best fit and was used for all subsequent analyses. The optimal number of bioregions (k) was determined with the optimal_phyloregion function of the phyloregion R package (7), testing solutions up to k = 30 and selecting the k that minimized an entropy-based stopping criterion. To improve topological robustness against ties in Pβsim values, the final regionalization was produced using the recluster.region function of the recluster R package (8), which repeats hierarchical clustering across 100 bootstrap phylogenetic trees and returns a consensus dendrogram weighted by bootstrap support. The maximum-clade-credibility tree (maxCladeCred; phangorn package; 9) was used as the reference tree for this procedure, and the clustering solution at the optimal k was extracted from the resulting consensus dendrogram.

Ordination and stability analysis. To visualize inter-regional relationships, hexagonal cells were aggregated to the bioregion level, and pairwise Pβsim was recomputed between all region pairs across the same 100 bootstrap trees; the per-pair median was used as the final inter-regional dissimilarity. Non-metric multidimensional scaling (NMDS) ordination was performed on this inter-regional distance matrix using the monoMDS function (vegan package; final stress = 0.274). To assess the consistency of region and realm assignments, the full clustering procedure was repeated with multiple independent seeds, and the results were consistent across runs.

Methods-specific References

1. H. Wickham, *ggplot2: Elegant Graphics for Data Analysis* (Springer-Verlag New York, 2016).
2. R. Wang, J. M. Kass, C. Chaudhary, E. P. Economo, B. Guénard, Global biogeographic regions for ants have complex relationships with those for plants and tetrapods. Nat. Commun. 15, 5641 (2024).
3. J. B. Dorey, et al., A globally synthesised and flagged bee occurrence dataset and cleaning workflow. Sci. Data 10, 747 (2023).
4. J. M. Kass, R. Muscarella, P. J. Galante, C. L. Bohl, G. E. Pinilla-Buitrago, R. A. Boria, et al., ENMeval 2.0: Redesigned for customizable and reproducible modeling of species' niches and distributions. Methods Ecol. Evol. 12, 1602-1608 (2021).
5. P. Henríquez-Piskulich, A. F. Hugall, D. Stuart-Fox, A supermatrix phylogeny of the world's bees (Hymenoptera: Anthophila). Mol. Phylogenet. Evol. 190, 107963 (2024).
6. A. Baselga, C. D. L. Orme, betapart: An R package for the study of beta diversity. Methods Ecol. Evol. 3, 808-812 (2012).
7. B. H. Daru, P. Karunarathne, K. Schliep, phyloregion: R package for biogeographical regionalization and macroecology. Methods Ecol. Evol. 11, 1483-1491 (2020).
8. L. Dapporto, M. Ramazzotti, S. Fattorini, G. Talavera, R. Vila, R. L. Dennis, recluster: An unbiased clustering procedure for beta-diversity turnover. Ecography 36, 1070-1075 (2013).
9. K. P. Schliep, phangorn: Phylogenetic analysis in R. Bioinformatics 27, 592-593 (2011).
